# Behavioral Effect of Plant Volatiles Binding to *Spodoptera littoralis* Larval Odorant Receptors

**DOI:** 10.3389/fnbeh.2018.00264

**Published:** 2018-11-12

**Authors:** Arthur de Fouchier, Xiao Sun, Gabriela Caballero-Vidal, Solène Travaillard, Emmanuelle Jacquin-Joly, Nicolas Montagné

**Affiliations:** Institut National de la Recherche Agronomique (INRA), Sorbonne Université, CNRS, IRD, UPEC, Université Paris Diderot, Institute of Ecology and Environmental Sciences of Paris, Paris and Versailles, France

**Keywords:** insect, olfaction, olfactory receptor, volatile organic compound, crop pest, caterpillar, Lepidoptera, Noctuidae

## Abstract

Phytophagous insects use volatile organic compounds (VOC) emitted by plants to orient towards their hosts. In lepidopteran pests, crop damages are caused by larval stages—the caterpillars—that feed extensively on leaves or other plant tissues. However, larval host plant choice has been poorly studied, and it is generally admitted that caterpillars feed on the plant where the female laid the eggs. The mobility of caterpillars has been generally overlooked even though several studies showed that they can orient towards odors and change host plant. Recently, a large number of odorant receptors (ORs) tuned to plant volatiles have been characterized in the model pest moth *Spodoptera littoralis* (Noctuidae). In the present work, we identified nine of these deorphanized ORs as expressed in *S. littoralis* caterpillars. In order to understand whether these ORs are involved in host searching, we tested the behavioral significance of their ligands using a larval two-choice assay. This OR-guided approach led to the identification of nine plant volatiles, namely 1-hexanol, benzyl alcohol, acetophenone, benzaldehyde, (Z)3-hexenol, (E)2-hexenol, indole, DMNT and (Z)3-hexenyl acetate, which are active on *S. littoralis* caterpillar behavior, increasing our knowledge on larval olfactory abilities. To further explore the link between OR activation and behavioral output induced by plant volatiles we used a modeling approach, thereby allowing identification of some ORs whose activation is related to caterpillar attraction. These ORs may be promising targets for future plant protection strategies.

## Introduction

Holometabolous insects are characterized by two mobile developmental stages with drastically different morphologies and physiologies. The larval stage constitutes a period of active feeding and growth, while the adult stage is a period devoted to reproduction and dispersal. Larvae and adults thus have different life styles, are not in competition for the same resources, and develop independent adaptations in response to different selective pressures. This distinction between adults and larvae is particularly striking in Lepidoptera. While larvae (or caterpillars) are actively feeding on their host plant, the adults generally live only a few days and feed on the nectar of flowers (Powell, 2009). Almost all plant species are damaged by caterpillars, many of which are pests of both crops and stored products (Stehr, 2009).

Host plant choice is a crucial task for phytophagous insects, and it is highly dependent on the sense of smell. The detection of plant-emitted volatile organic compounds (VOC) has been the subject of intense research, notably in crop pest insects (Bruce and Pickett, 2011; Bruce et al., 2015). In a number of lepidopteran pests, VOCs have been identified as attractants towards host plants, as repellents towards non-host or damaged plants or as oviposition stimulants (Saveer et al., 2012; Borrero-Echeverry et al., 2015). However, despite the impact of caterpillars on crop production, most studies focused on the adults and little is known about larval olfaction. A well-admitted theory, referred as “mother knows best,” assumes a strong selective pressure for females to lay their eggs on the plant where the larvae will have the highest performance (Jaenike, 1978; Carrasco et al., 2015). However, in some species it has been demonstrated that the caterpillars can leave the plant on which they hatched to select another host plant (Soler et al., 2012; Gamberale-Stille et al., 2014). Consistently, caterpillars exhibit attraction or repulsion behaviors towards VOCs of ecological significance (Carroll and Berenbaum, 2002; Huang and Mack, 2002; Singh and Mullick, 2002; Carroll et al., 2006, 2008; Castrejon et al., 2006; Becher and Guerin, 2009; Mooney et al., 2009; Piesik et al., 2009; Poivet et al., 2012; Zhu et al., 2016; Di et al., 2017) and are even able to perform associative learning (Blackiston et al., 2008; Salloum et al., 2011). This indicates that olfaction may play a more prominent role than initially expected in host plant choice of caterpillars, which could lay foundation for the development of novel pesticide-free strategies for fighting against those insects.

The peripheral olfactory system of caterpillars is generally composed of three olfactory sensilla located on the antennae, and four to five olfactory sensilla located on the maxillary palps (Grimes and Neunzig, 1986; Laue, 2000; Vogt et al., 2002; Roessingh et al., 2007; Poivet et al., 2012; Zielonka et al., 2016). These sensilla house the olfactory sensory neurons that express transmembrane odorant receptor (OR) proteins, which bind odorants and allow signal transduction (Leal, 2013). The repertoires of ORs expressed in caterpillar tissues have been identified only in a few species, such as the silkworm *Bombyx mori* (Tanaka et al., 2009), the cotton bollworm *Helicoverpa armigera* (Di et al., 2017) and the cotton leafworm *Spodoptera littoralis* (Poivet et al., 2013). In this latter species, 15 ORs (further referred as SlitORs) tuned to plant VOCs have been recently deorphanized (de Fouchier et al., 2017), i.e., their ligands have been identified (Supplementary Figure S1). These VOCs are mainly short-chain alcohols, aldehydes or esters (also referred as green leaf volatiles, abundantly released from damaged leaves), aromatics and terpenes (most of them being ubiquitous odorants, present in high amounts in floral bouquets). However, the effect of these SlitOR ligands on the behavior of *S. littoralis* larvae remains largely unknown. Among them, only 1-hexanol (a green leaf volatile) has been shown to be attractive at high dose toward 2nd and 3rd-instar larvae (Rharrabe et al., 2014).

In the present work, we first re-examined the expression pattern of the 15 deorphanized SlitORs in adult and larvae olfactory organs, and identified nine as expressed at the larval stage. We then used a simple bioassay to carry out a systematic behavioral analysis of 14 VOCs previously identified as ligands of these nine SlitORs. Using this OR-guided approach, we found 1-hexanol, benzyl alcohol, acetophenone, benzaldehyde, (Z)3-hexenol, (E)2-hexenol, indole, DMNT and (Z)3-hexenyl acetate as active on the behavior of *S. littoralis* caterpillars, increasing our knowledge on larval olfactory abilities. Building on the results of these behavioral assays and on our previous knowledge of SlitOR response spectra (de Fouchier et al., 2017), we used a modeling approach in order to identify possible correlations between the activation of SlitORs and the behavioral response of caterpillars. By doing so, we highlighted ORs whose activation may be critical for larval attraction towards plant volatiles.

## Materials and Methods

### Insects and Chemicals

*S. littoralis* larvae were reared on a semi-artificial diet (Poitout and Bues, 1974) at 22°C, 60% relative humidity and under a 16 h light: 8 h dark cycle. The panel of odorants tested was composed of 14 synthetic molecules (Supplementary Table S1) previously shown to be active on SlitORs expressed at the larval stage (de Fouchier et al., 2017). Odorants were diluted in paraffin oil (Sigma-Aldrich, St. Louis, MO, USA), except indole that was diluted in hexane (Carlo-Erba Reagents, Val de Reuil, France). The odorants were used at concentrations of 100, 10, 1, 0.1 or 0.01 μg/μl.

### RNA Isolation and Reverse-Transcription PCR

Fifty *S. littoralis* male and female adult antennae and 50 pairs of 4th-instar larvae antennae and maxillary palps were dissected and immediately placed in TRIzol™ Reagent (Thermo Fisher Scientific, Waltham, MA, USA) for total RNA extraction. After isolation using phenol-chloroform, RNA was purified using the RNeasy Micro Kit (Qiagen, Venlo, Netherlands), including a DNase I treatment. RNA purity and quantity were measured on a NanoDrop™ ND-2000 spectrophotometer (Thermo Fisher Scientific). cDNA synthesis was performed using 1 μg of total RNA as template, with the iScript Reverse Transcription Supermix (BioRad, Hercules, CA, USA). PCRs were performed using the LightCycler^®^ 480 SYBR Green I Master mix (Roche, Basel, Switzerland) under the following conditions: 95°C for 5 min, followed by 40 cycles of denaturation (95°C for 10 s), hybridization (58–62°C—depending on primer pairs—for 15 s) and elongation (72°C for 15 s). Primer pairs were designed from SlitOR nucleotide sequences using Primer3Plus^1^. All primer sequences, annealing temperatures and expected product sizes are listed in Supplementary Table S2. Orco, the obligatory OR co-receptor (Malpel et al., 2008; Leal, 2013), was used as control for the four tissues. For each amplification, negative controls consisted of amplifications run on DNase-treated RNAs and water templates. The amplification products were loaded on 1.5% agarose gels and visualized using GelRed™ Nucleic Acid Gel Stain (Biotium, Fremont, CA, USA). Tissue dissections, RNA extractions and RT-PCR experiments were repeated three times at different periods, to serve as biological replicates.

### Behavioral Experiments

**Figure 1:**
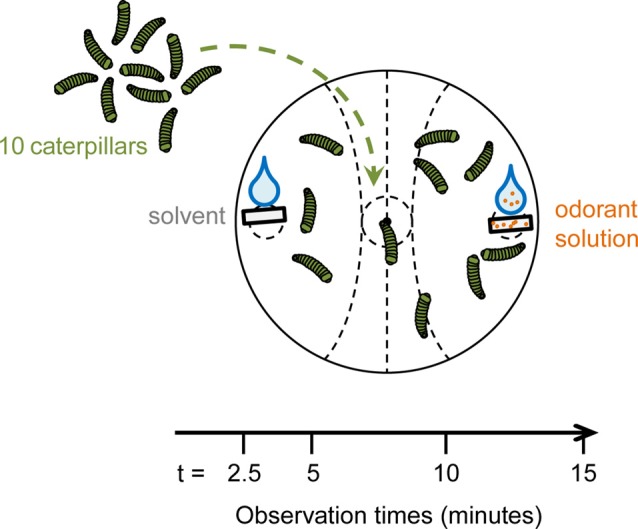
Schematic of the behavior assay design. Ten 3rd and 4th-instar caterpillars were put in the center of a Petri dish after being starved for 16–22 h. On one side of the dish, a filter paper with 10 μl of an odorant solution was placed. Another filter paper with 10 μl of solvent was put at the opposite side of the dish. The numbers of caterpillars in the different zones were recorded at 2.5, 5, 10 and 15 min. The preference index (PI), ranging for 1 (attraction) to −1 (repulsion), was calculated for each observation time.

Two-choice behavioral assays were performed using *S. littoralis* 3rd and 4th-instar larvae, starved for 16–22 h prior to experiments. The behavioral assay consisted in placing 10 caterpillars in the center of a Petri dish. Filter papers were placed at two opposite sides of the dish. One was loaded with 10 μl of an odorant solution and the other with 10 μl of the corresponding solvent. Each odorant concentration was tested 10–15 times. For each experiment, 10 Petri dishes (containing 10 different odorants) and one control dish with solvent on both sides were recorded during 15 min. In each dish, two zones were defined around the filter papers, an “odorant” zone and a “solvent” zone (the layout of the zones are visible in Figure 1). The number of caterpillars in each zone was counted 2.5, 5, 10 and 15 min after the beginning of the experiment.

### Data Analysis and Modeling

For each time point, a preference index (PI) was calculated using the following formula:

$$\text{PI}=({\text{N}}_{\text{odorant}}-{\text{N}}_{\text{solvent}})/({\text{N}}_{\text{total}})$$

N_odorant_ being the number of larvae in the odorant zone, N_solvent_ being the number of larvae in the solvent zone and N_total_ being the total number of larvae in the assay. As this PI varies between −1 and 1, a positive value means that the odorant is attractive and a negative value indicates repellency. To test for the statistical significance of the observed PI, we compared the value to a theoretical value of 0 with a Wilcoxon two sided unpaired test using R (Package stats version 3.3.2).

In order to compare observed PIs with responses of the SlitORs (in spikes.s^−1^) when expressed in the *Drosophila* empty neuron system (de Fouchier et al., 2017), we performed multiple linear regressions using the “step” and “lm” function of R (Package stats version 3.3.2). To obtain the most efficient equation, we performed stepwise linear regressions relating PI with all possible interactions between the larval SlitOR responses (SlitOR7, 14, 19, 24, 25, 27, 28, 29 and 31). As odorant stimulus quantities used in electrophysiology experiments cannot be directly related to quantities used in the present behavior experiments, we built models for different electrophysiology-behavior odorant quantity relationships (1:1, 1:1/10, 1:1/100 and 1:1/1,000). We selected the equation with the highest *R*^2^ and refined it performing another stepwise multiple linear regression. This model relates the PI with all the interactions between the factors with an impact significantly different from zero (Pr(>*t*) *p* ≤ 0.05) in the previously selected model. To further simplify the model, we performed a last multiple linear regression relating PI with only additive interactions of the previously used variables.

We also built some models to further test the importance of the different SlitORs in predicting larval PI. One using all possible interactions between the responses of SlitOR14, 19, 28, 29 and 31, and four other models using linear regressions of the PI explained by the response from only SlitOR7, 24, 25 or 27.

## Results

### Expression of SlitORs at the Larval Stage

**Figure 2:**
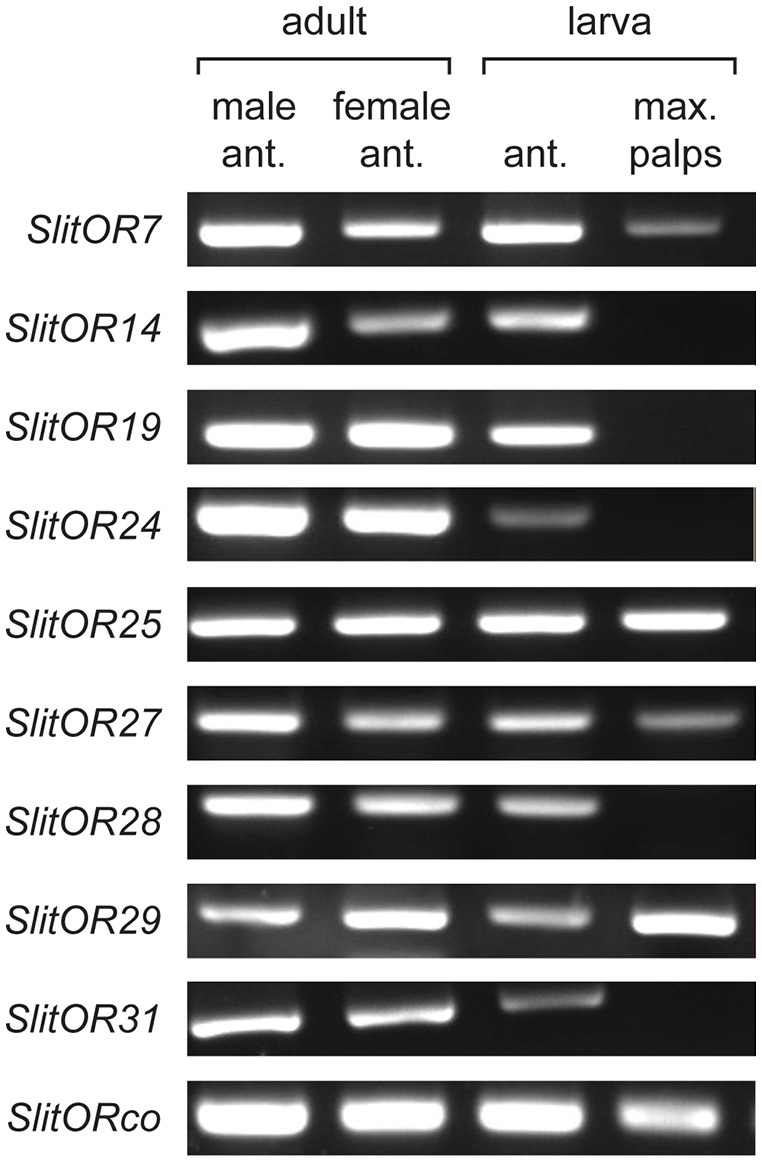
Tissue-specific expression of larval *S. littoralis* odorant receptors (ORs) identified by RT-PCR. Each RT-PCR was repeated three times on three separate RNA extractions. Only SlitORs found to be expressed in larval antennae or maxillary palps in the three replicates are shown.

The expression pattern of 15 previously deorphanized SlitORs in male and female adult antennae, larval antennae and larval maxillary palps (4th-instar larvae) was re-investigated using RT-PCR. As found previously, all SlitORs were expressed in male and female antennae. Among them, nine SlitORs were also expressed in larval tissues (Figure 2). Five ORs were expressed in larval antennae (SlitOR14, 19, 24, 28 and 31), and four ORs were expressed in both larval antennae and maxillary palps (SlitOR7, 25, 27, 29). Altogether, these nine ORs were previously found to detect 20 plant VOCs (Supplementary Figure S1) among a panel of 50 molecules from different chemical classes, when expressed in the *Drosophila* empty neuron system (de Fouchier et al., 2017). We then selected a panel of 14 of these odorants, chosen based on the distinct OR activation patterns they elicit, in order to test their effect on larval behavior.

### Behavior of *S. littoralis* Caterpillars Toward SlitOR Ligands

**Figure 3:**
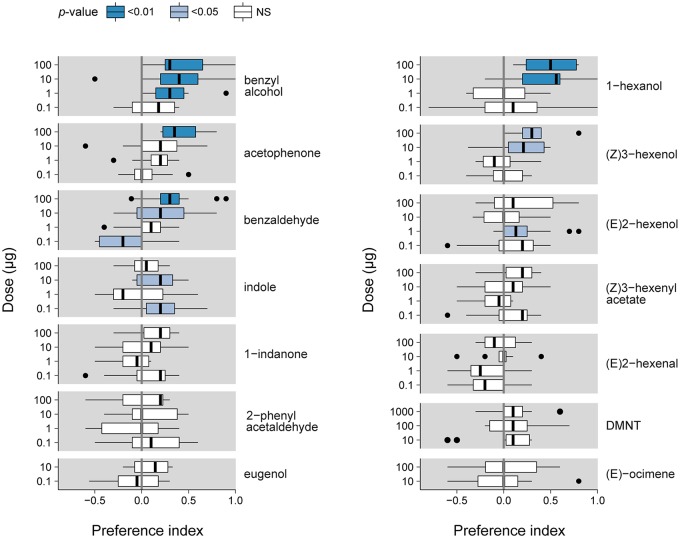
*S. littoralis* larval PI measured 2.5 min after exposure to different odorant stimuli. Box plots show the median PI and the 25th and 75th percentiles (*n* = 8–15). Outliers are indicated with black dots. *p*-values are indicated using a color code (Wilcoxon test).

We assessed the valence of plant VOCs for *S. littoralis* caterpillars by describing their repartition in a two-choice bioassay (Figure 1) using a PI over a period of 15 min. Figure 2 reports the PIs measured at 2.5 min for the different VOCs at different doses. PIs measured for other time points are presented in Supplementary Figure S2. For 2-phenyl acetaldehyde, 1-indanone, (E)-ocimene and eugenol, we observed no significant attraction (PI > 0) or repulsion (PI < 0), at any dose and any time. Benzyl alcohol, acetophenone, benzaldehyde, indole, 1-hexanol, (Z)3-hexenol and (E)2-hexenol were attractive at least at one dose, with the highest PI measured at 2.5 min (Figure 3). 1-hexanol displayed the strongest attraction, with a mean PI of 0.50 at 100 μg, and 0.44 at 10 μg. Benzyl alcohol was attractive over the wider range of doses, from 100 down to 1 μg per filter paper. Benzaldehyde elicited attraction at 100 and 10 μg, and acetophenone only at 100 μg. Indole was attractive at 10 and 0.1 μg only and (E)2-hexenol was attractive only at 1 μg. For most of these VOCs, the PI tended to decrease over time (Supplementary Figure S2), which suggests that sensory adaptation occurred. The only stimulus that remained attractive over time was acetophenone, when presented at the highest dose (100 μg). (Z)3-hexenyl acetate differed from the previous VOCs as doses of 100 and 10 μg were found to be attractive after 5 min of experiment, and not after 2.5 min (Supplementary Figure S2).

At 2.5 min, benzaldehyde (at 0.1 μg) was the only VOC found to be repulsive (Figure 3). (Z)3-hexenyl acetate (1 μg) was repulsive after 5 min, and (E)2-hexenal and DMNT also induced a negative PI (for 0.1 and 100 μg, respectively) at 15 min of observation (Supplementary Figure S2).

### Modeling of the Relationship Between SlitOR Activation and Behavioral Activity Induced by Their Ligands

We next aimed to identify which of the SlitORs could be linked to attraction or repulsion towards plant VOCs. To assess the correlation between the valence of odorants and their activation pattern of ORs, we built models relating caterpillar PIs measured here with larval SlitOR responses to the same odorants (previously characterized in de Fouchier et al., 2017). We used stepwise multiple linear regressions, taking into account all possible interactions between the variables. The equations of the first models built are available in Supplementary Datasheet 2. The multiple linear regression giving the highest adjusted *R*^2^ (0.6861) was the one using a 1:1 relationship between quantities used in behavior and electrophysiology experiments (Table 1).

**Table 1:** SlitOR/behavior multiple linear regression model statistics.

Model	Adjusted *R*^2^	Residual standard error	*F*-test	Shapiro test
Model 1:1	0.6861	0.09647	***	***
Model 1:1/10	0.6225	0.1048	***	NS
Model 1:1/100	0.5795	0.1106	***	*
Model 1:1/1000	0.3061	0.142	***	NS
Refined 1:1 model	0.6366	0.1038	***	**
Minimal 1:1 model	0.6115	0.1073	***	NS

Statistics associated with the models of S. littoralis caterpillars PIs. The Shapiro Test column indicates the p-value of a normality test for the distribution of the model residuals. ***p ≤ 0.001, **p ≤ 0.01, *p ≤ 0.05, NS: p > 0.05.

**Figure 4:**
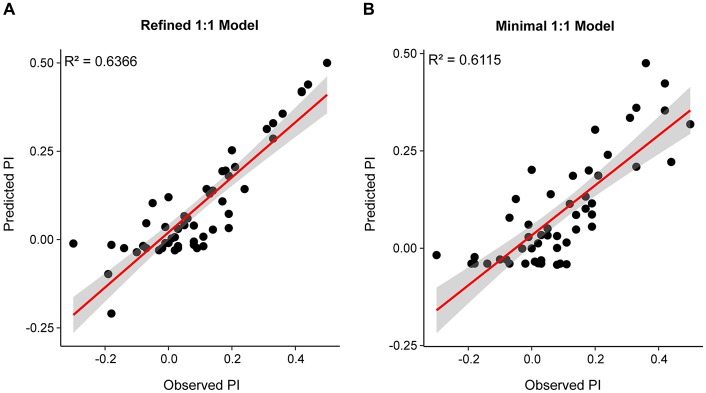
Predicted PI plotted as a function of the observed PI for the refined **(A)** and minimal models **(B)**. Red lines depict the linear trend while the overlaying gray band is the SE for the fit.

To identify the SlitORs whose activation is the most critical to the valence of plant odorants for caterpillars, we refined the equation of the 1:1 model. For this, we performed stepwise multiple linear regressions taking into account all possible interactions between the factors with an effect significantly different from zero in the 1:1 model (Pr(>*t*) *p* ≤ 0.05). This model was able to describe the variation of PIs from the responses of 5 SlitORs (SlitOR7, 14, 24, 25 and 27; *F*-Test, *p* ≤ 0.001, *R*^2^ = 0.6366, Table 1, Figure 4A and Supplementary Figure S3). The equation of the refined model is given in Supplementary Datasheet 2. The intercept value of this model was not different from 0 (Pr(>*t*) *p* ≥ 0.05), which predicts that an absence of SlitOR activation would result in an absence of behavioral output. In this refined model, activation of SlitOR24 was predicted to have a positive effect by itself on PIs (Pr(>*t*) *p* ≤ 0.05), whereas activations of SlitOR7, 25 and 27 were predicted to have an effect on PIs only through OR co-activation. SlitOR14 associated coefficients were not different from 0 (Pr(>*t*) *p* ≥ 0.05).

As the refined model had a complicated equation (20 terms), we then built a simpler model to predict the behavior using only additive interactions. The equation of this minimal model is:

PI=a+b×SlitOR7+c×SlitOR24+d×SlitOR25+e×SlitOR27

with *SlitORx* as the ORx responses to the considered odorant in spikes.s^−1^ and *a-e* as coefficients. The values of these coefficients (available in Supplementary Datasheet 2) were all different from 0 (Pr(>*t*) *p* ≤ 0.05), except for the intercept. The *R*^2^ value for this model was 0.6115 (Table 1, Figure 4B and Supplementary Figure S3), which is comparable to the performances of the refined 1:1 model. SlitOR24 had the highest coefficient (2.6070 × 10^−3^, *p* ≤ 0.001), which further supports a link between this receptor and neuronal circuits driving attraction in *S. littoralis* larvae. It is interesting to note that the coefficient associated with SlitOR7 was negative (−5.0528 × 10^−3^, *p* ≤ 0.05). This predicts that activation of SlitOR7 has a negative effect of the PI of *S. littoralis* caterpillars.

To further confirm the importance of those four SlitORs for models performance in predicting the observed PI, we tried to build a model using all interactions between all the SlitORs except SlitOR7, 24, 25 and 27. The stepwise multiple linear regressions method was unable to produce a model from these variables, thus highlighting the importance of these receptors for the response of caterpillars to the VOCs tested. We also built models using the responses from only SlitOR7, 24, 25 or 27. The *R*^2^ values for these models were respectively: 0.15, 0.48, 0.19 and 0.04. The values of the coefficients of the intercept and of the SlitOR response were different from 0 (Pr(>*t*) *p* ≤ 0.05), except for the intercept of the model based on SlitOR24. These observations support that SlitOR24 is the most important receptor to predict the PI observed for the plant volatiles we tested.

## Discussion

Building upon the previous identification of ligands for a large number of *S. littoralis* ORs, we aimed at identifying behaviorally active odorants for caterpillars, which are pests feeding on a wide range of plants, notably economically important ones (Salama et al., 1971; Cabello, 1989; Thöming et al., 2013; von Mérey et al., 2013; Proffit et al., 2015). Nine *S. littoralis* ORs were confirmed to be expressed in larval chemosensory organs, namely the antennae and the maxillary palps. Our “OR-guided” strategy, by which we tested molecules active on these larval SlitORs, appeared as a good strategy as we could identify plant VOCs being behaviorally active when presented alone, most of them being attractive to caterpillars. Following that work, it will be of interest to test the effect of blends of these VOCs. It has been shown in *H. armigera* that a mixture of the best ligands of four ORs was the most attractive stimulus for first-instar larvae (Di et al., 2017), and one would expect that the same holds true for *S. littoralis*.

Our study complements a former study (Rharrabe et al., 2014) that investigated 11 odorants commonly emitted by plants, identifying only a small part of them as behaviorally active. In this previous work, eugenol was found to be repellent and 1-hexanol attractive. Here, attraction towards 1-hexanol could be reproduced in our assay but eugenol was inactive. This discrepancy could be explained by the fact that odorants and controls were presented together with food pellets in the aforementioned study while we used only filter papers as odor source. Hence, it is likely that repellent VOCs for *S. littoralis* caterpillars may be identified only when given the choice between food sources (or food odors) with or without the VOC.

Another interesting difference between these two types of behavioral assays is that the presence of food will make the larvae stay on the food source once they have made a choice. In our experiments, larvae resumed foraging after their initial choice, which enabled to observe a decrease of the PI in most cases, likely due to sensory adaptation. Another possible explanation for this PI decrease would be that the volume of the Petri dish has been rapidly saturated with the odor, leading to a loss of the odor gradient necessary for larval orientation.

A similar OR-guided approach was recently used on another species of pest caterpillars, *H. armigera*, and led to the identification of several OR ligands that were active on the behavior of first-instar larvae (Di et al., 2017). Even if *S. littoralis* and *H. armigera* both belong to the same family (Noctuidae) and are both highly polyphagous herbivores, their larval OR repertoires seem to differ drastically. Indeed, the orthologs of only three of the nine larval SlitORs were also found to be expressed in *H. armigera* larvae (Di et al., 2017). The same holds true when comparing with the more distantly related species *B. mori* (Tanaka et al., 2009). Accordingly, a limited number of odorants identified as active on *S. littoralis* larvae are also active on other species, and vice versa.

The most attractive VOC (i.e., with the highest PI) was 1-hexanol, an ubiquitous plant volatile (Knudsen et al., 2006), which has been observed to be attractive for caterpillars of the Tortricidae *Lobesia botrana* (Becher and Guerin, 2009). Among other attractive compounds for *S. littoralis* larvae, (Z)3-hexenol was also observed to be attractive to *L. botrana* and *H. armigera* (Di et al., 2017), but not to *B. mori* (Tanaka et al., 2009). (Z)3-hexenyl acetate is a volatile released by plants that suffered attacks from insects and it has been reported to serve as a chemical message between plants (Frost et al., 2008; Helms et al., 2014). It has been observed to be attractive for the larvae of *S. littoralis* (this study), *H. armigera*, *L. botrana*, and *B. mori*. This suggests that (Z)3-hexenyl acetate is an important cue for a large spectrum of lepidopteran species. However, at a lower dose (1 μg), it is also the most repulsive VOC for *S. littoralis* caterpillars. Further experiments specially designed for the identification of repellents would be necessary to confirm this repulsive effect, but *S. littoralis* might use (Z)3-hexenyl acetate to detect and avoid damaged plants. Indeed, it has been demonstrated previously that *S. littoralis* larvae are able to discriminate between different leaves of a host plant and show a preference for young leaves, this preference being modified by herbivore damage (Anderson and Agrell, 2005). (Z)3-hexenyl acetate is detected via the activation of several ORs (de Fouchier et al., 2017). Their differential activation pattern relative to the dose may encode the concentration, as previously hypothesized for pheromone receptors detecting the same pheromone component in adults (de Fouchier et al., 2015).

From the comparison of behavior results with our previous results on SlitOR deorphanization (de Fouchier et al., 2017), we built models that can predict PI values for odorants based on their OR activation pattern. Results of this modeling approach suggest that larval attraction depends on the activation of a particular subset of ORs (i.e., circuit-based) rather than on the summed response of the entire OR repertoire. This will be possible to confirm this hypothesis only when the complete larval OR repertoire will be characterized. In *D. melanogaster*, similar linear regression-based approaches allowed to predict larval behavior from the responses of only five ORs (Kreher et al., 2008). Still in *D. melanogaster*, a strong link has been identified between larval attraction and activation of two larval ORs, DmelOR42a and DmelOR42b (Kreher et al., 2008; Asahina et al., 2009; Grewal et al., 2014). Here, models supported that SlitOR24, 25 and 27 are involved in pro-attraction neuronal circuits, while SlitOR7 activation would antagonize attraction. Activation of the first three receptors, especially SlitOR24, seems to be sufficient to trigger attraction of *S. littoralis* toward different concentrations of odorants. This will need further experimental validation, notably by identifying new ligands for these receptors and testing their behavioral effect, but it could be a promising way to identify new compounds that could impact the behavior of this important crop pest.

## Author Contributions

AF, EJ-J and NM designed the study. AF, XS and ST performed behavioral experiments. GC-V performed molecular biology experiments. AF performed modeling experiments. AF, EJ-J and NM wrote the manuscript, with input from all authors.

## Conflict of Interest Statement

The authors declare that the research was conducted in the absence of any commercial or financial relationships that could be construed as a potential conflict of interest.

## Footnotes

^1^http://www.bioinformatics.nl/cgi-bin/primer3plus/primer3plus.cgi
