# Supplementary material for: Behavioral Effect of Plant Volatiles Binding to Spodoptera littoralis Larval Odorant Receptors
Source: Front Behav Neurosci. 2018 Nov 12;12:264. doi: 10.3389/fnbeh.2018.00264 (PMC6240680; doi:10.3389/fnbeh.2018.00264)

Supplementary Material

Behavioral effect of plant volatiles binding to *Spodoptera littoralis* larval odorant receptors

Arthur de Fouchier[#], Xiao Sun[‡], Gabriela Caballero-Vidal, Solène Travaillard[¶], Emmanuelle Jacquin-Joly & Nicolas Montagné[*]

Inra, Sorbonne Université, CNRS, IRD, UPEC, Université Paris Diderot, Institute of Ecology and Environmental Sciences of Paris, Paris and Versailles, France

[#] present address: Laboratoire d'Ethologie Expérimentale et Comparée (LEEC), Université Paris 13, Sorbonne Paris Cité, Villetaneuse, France
[‡] present address: School of Life Sciences, Henan University, Kaifeng, Henan, China
[¶] present address: Aix-Marseille Université, CNRS, Institut de Biologie du Développement de Marseille, Marseille, France

*** Correspondence:**
Nicolas Montagné
nicolas.montagne@sorbonne-universite.fr

Supplementary Table S1. Synthetic volatile organic compounds used in behavioral assays.

Compound	CAS number	Provider	Purity
benzyl alcohol	100-51-6	Aldrich	99
acetophenone	98-86-2	Acros	99
benzaldehyde	100-52-7	Aldrich	99.5
Indole	120-72-9	Aldrich	99
1-indanone	83-33-0	Aldrich	99
2-phenyl acetaldehyde	122-78-1	Aldrich	98
Eugenol	97-53-0	Aldrich	98
1-hexanol	111-27-3	Aldrich	98
(Z)3-hexenol	928-96-1	Aldrich	98
(E)2-hexenol	928-97-2	Aldrich	96
(Z)3-hexenyl acetate	3681-71-8	Aldrich	98
(E)2-hexenal	6728-26-3	Aldrich	98
(E)-ocimene	3779-61-1	Aldrich	65 (E)
(E)-4,8-dimethyl-1,3,7-nonatriene (DMNT)	19945-61-0	Gift from Pr. Wittcko Francke, Hamburg	99

Supplementary Table S2. Primers used in RT-PCR experiments.

Odorant receptor	Forward primer sequence	Reverse primer sequence	T_m (°C)	Product size (bp)
SlitOR3	GTATGGGATGCTGGTGAGAGAAG	AGTGGATTGAAGACCTGGATATGC	58	163
SlitOR4	GCGCTTCAAGAACTGACGGCTAT	AACCGCAACAGTACACTGCCAT	60	427
SlitOR7	CCTTCCTATCGATGGCTCTG	CCCAGGTACCACTTGCAGTT	60	115
SlitOR14	CGTCATCACCCACAACCTCAC	CCCAATAGTCACCCAGCCAAAG	58	196
SlitOR17	GTAGCGATCGGTAACACAACAAT	CGAGCTCTCCACTGTTACTTCAT	60	414
SlitOR19	AAACGTGACTCCGTGAGCTT	CCGCCATCAACGTATTTTCT	62	148
SlitOR24	CGCATCCGTTTATCGACTTT	CAAACCAGACCACAAGAGCA	60	116
SlitOR25	AGCTTTCTGTTCCTGGCGTA	ATGATGGTAGACCGCACTCC	62	186
SlitOR27	ACCAAATTGGCGTTTCTGTC	ATGGTACAGTTGGGGGTTGA	60	80
SlitOR28	TGTAACTGGCGAGGGAAATCAC	GCTCTATATGGCTGCGGTTGG	58	133
SlitOR29	CGTCATCACCCACAACCTCAC	CCCAATAGTCACCCAGCCAAAG	58	196
SlitOR31	TTGGGGAAGCAAACTGCCTTCA	GAATCTTGGCTTGCGCATAGAACG	60	379
SlitOR32	TCTGAATAGGGCGAAGTTTGTAA	TGTGTAGGTCTTCACTCGTAGCA	60	944
SlitOR35	TGCGACCTGCCGACTATG	CTCCTCACGAACACGAACC	53	179
SlitOR36	GTCTCCATACTCCTGAGGGTTCT	GCTGCAAAAATGTATTCTCCAAC	60	904

Supplementary Figure S1. Heat map summarizing the mean responses of the 9 larval SlitORs to 100 µg of plant VOCs when expressed in the *Drosophila* empty neuron system (adapted from de Fouchier et al., 2017). Responses are color-coded according to the scale on the right (values are spikes.s^{-1}).

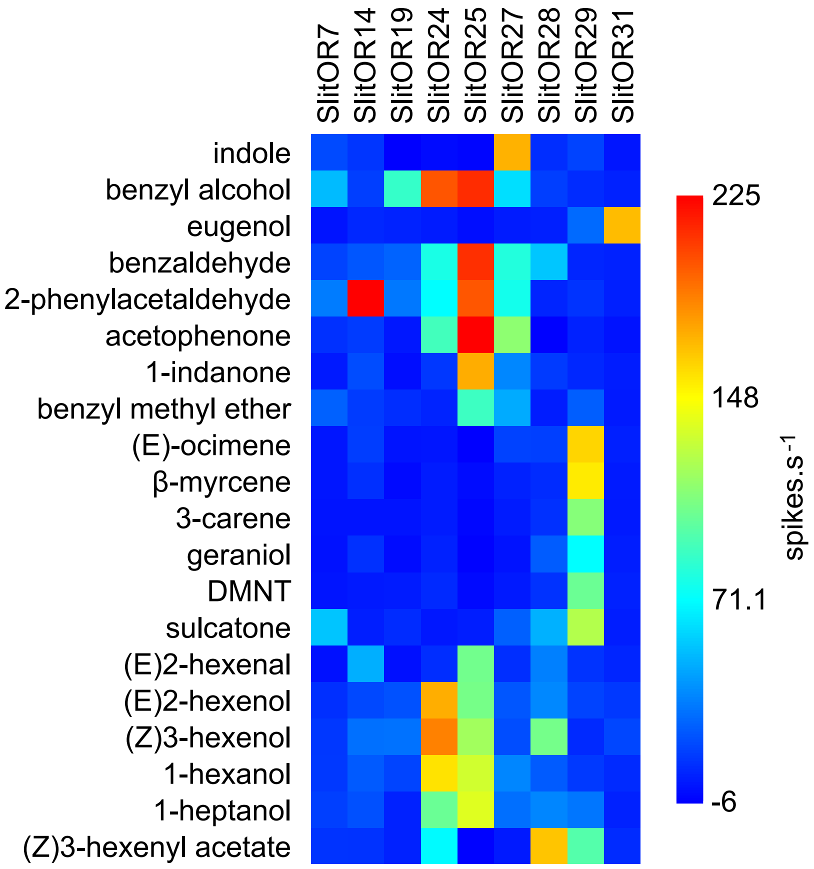

Supplementary Figure S2. *S. littoralis* mean preference index (PI) measured for different doses of plant VOCs after 2.5, 5, 10 and 15 minutes of experiment. Error bars indicate s.e.m. (n = 8-15). *: $p \leq 0.05$, **: $p \leq 0.01$, ***: $p \leq 0.001$ (Wilcoxon test).

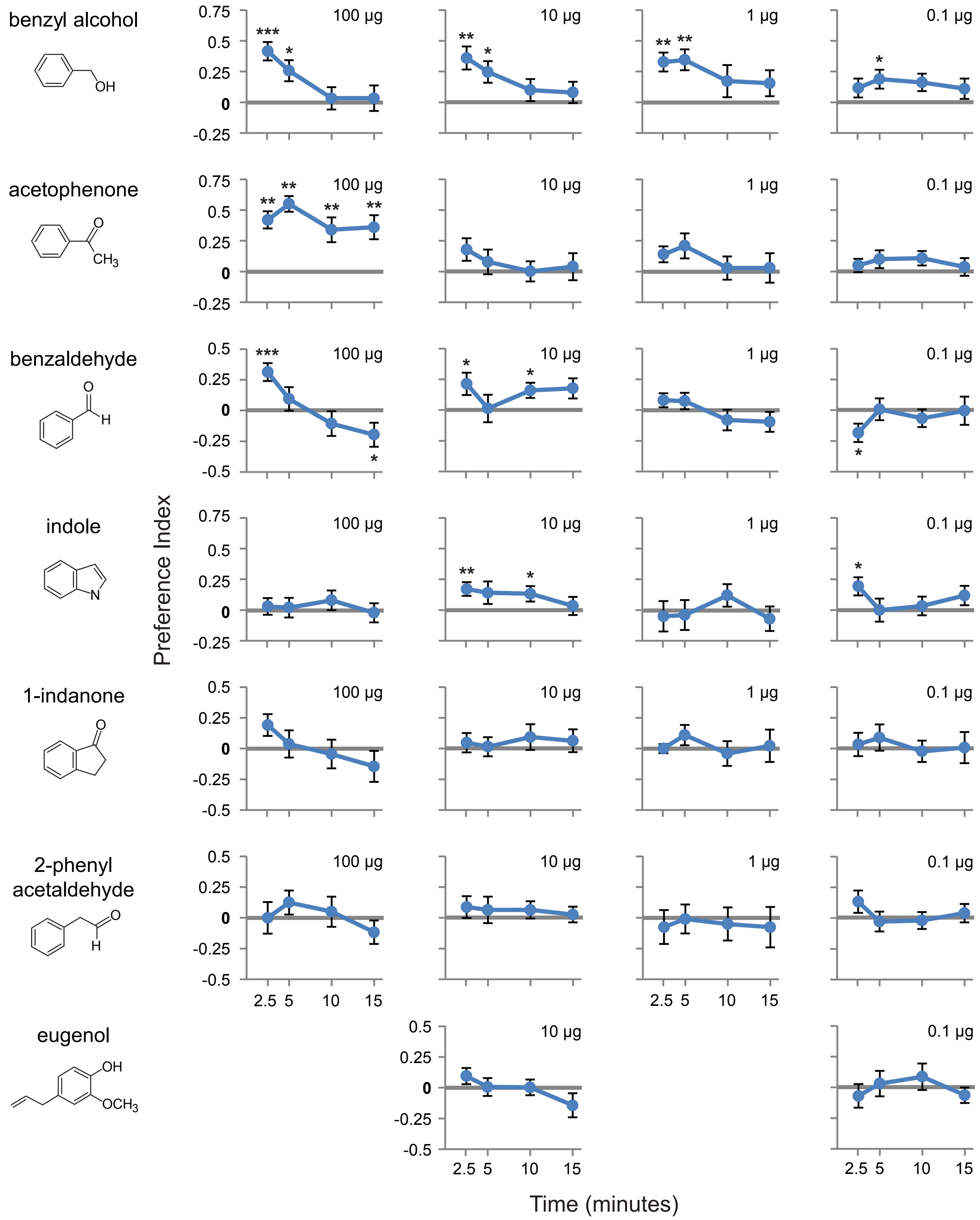

Supplementary Figure S2. continued

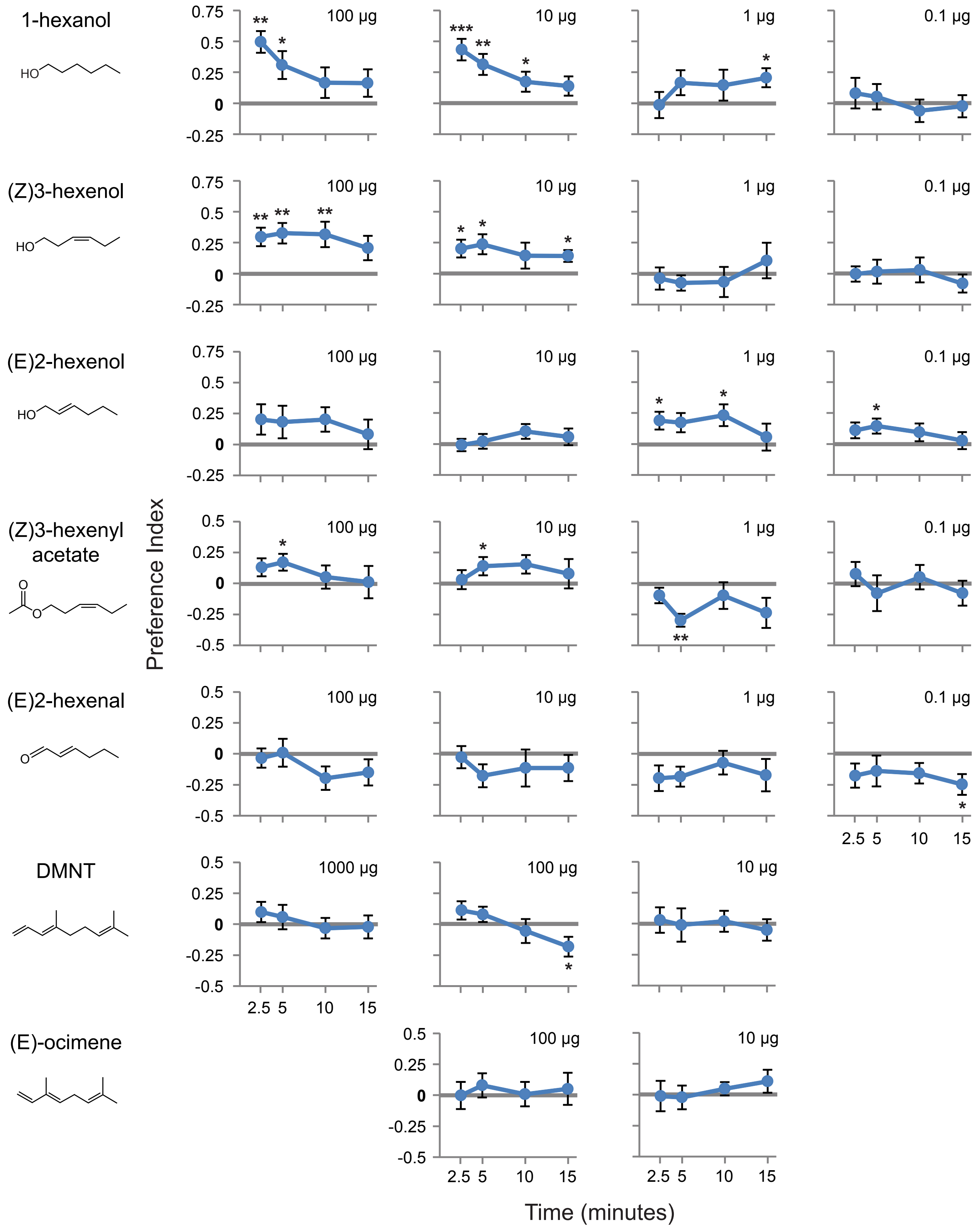

Supplementary Figure S3. *S. littoralis* mean preference index (PI) observed (green) or predicted from the refined (orange) or minimal (purple) models for different doses of plant VOCs.

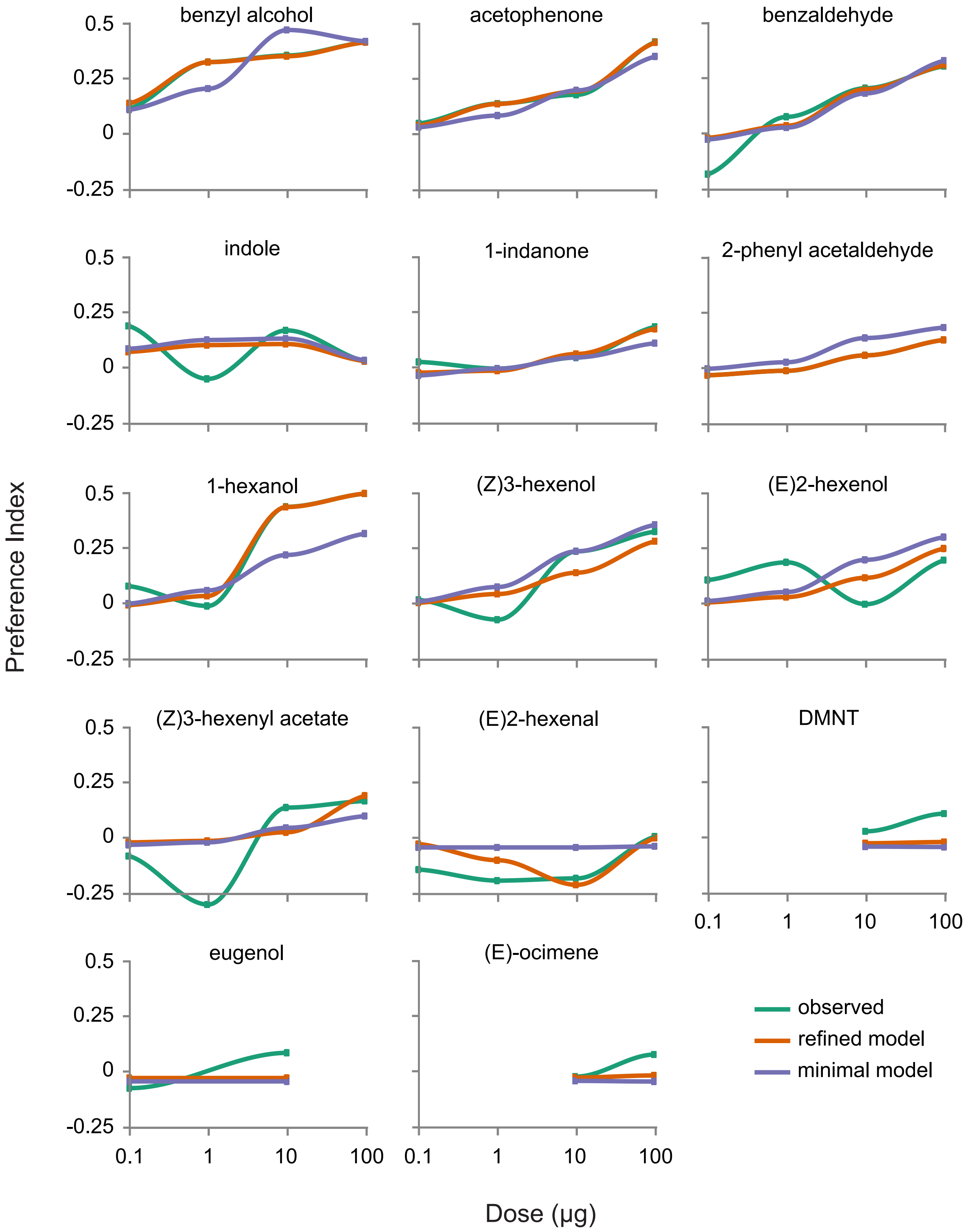

Supplement: Supplementary file 1 [file Data_Sheet_1.PDF]
