# Supplementary material for: Behavioral Effect of Plant Volatiles Binding to Spodoptera littoralis Larval Odorant Receptors
Source: Front Behav Neurosci. 2018 Nov 12;12:264. doi: 10.3389/fnbeh.2018.00264 (PMC6240680; doi:10.3389/fnbeh.2018.00264)
Supplement: Supplementary file 2 [file Data_Sheet_2.docx]

1:1 Model :

Formula :

PI = SlitOR7 + SlitOR14 + SlitOR19 + SlitOR24 + SlitOR25 + SlitOR27 + SlitOR28 + SlitOR29 + SlitOR7:SlitOR14 + SlitOR7:SlitOR19 + SlitOR14:SlitOR19 + SlitOR7:SlitOR24 + SlitOR14:SlitOR24 + SlitOR7:SlitOR25 + SlitOR14:SlitOR25 + SlitOR24:SlitOR25 + SlitOR7:SlitOR27 + SlitOR14:SlitOR27 + SlitOR19:SlitOR27 + SlitOR24:SlitOR27 + SlitOR25:SlitOR27 + SlitOR7:SlitOR28 + SlitOR24:SlitOR28 + SlitOR25:SlitOR28 + SlitOR27:SlitOR28 + SlitOR7:SlitOR29 + SlitOR24:SlitOR29 + SlitOR28:SlitOR29 + SlitOR7:SlitOR14:SlitOR24 + SlitOR7:SlitOR24:SlitOR25 + SlitOR7:SlitOR25:SlitOR27 + SlitOR24:SlitOR25:SlitOR27 + SlitOR7:SlitOR24:SlitOR28 + SlitOR24:SlitOR27:SlitOR28 + SlitOR25:SlitOR27:SlitOR28 + SlitOR24:SlitOR28:SlitOR29

Coefficients:

Estimate Std. Error t value Pr(>|t|)

(Intercept) 1.706e-02 3.842e-02 0.444 0.6633

SlitOR7 6.854e-01 4.129e-01 1.660 0.1177

SlitOR14 -9.567e-03 4.007e-03 -2.388 0.0306 *

SlitOR19 9.644e-03 1.332e-02 0.724 0.4801

SlitOR24 5.664e-04 1.106e-03 0.512 0.6161

SlitOR25 -2.678e-03 3.365e-03 -0.796 0.4384

SlitOR27 4.851e-04 4.150e-04 1.169 0.2607

SlitOR28 -4.770e-03 4.217e-03 -1.131 0.2758

SlitOR29 -2.236e-04 6.858e-04 -0.326 0.7489

SlitOR7:SlitOR14 -2.126e-02 8.090e-03 -2.628 0.0190 *

SlitOR7:SlitOR19 1.822e-01 1.359e-01 1.341 0.1999

SlitOR14:SlitOR19 6.849e-02 7.885e-02 0.869 0.3987

SlitOR7:SlitOR24 -4.229e-03 2.318e-03 -1.824 0.0881 .

SlitOR14:SlitOR24 7.725e-04 3.462e-04 2.231 0.0413 *

SlitOR7:SlitOR25 -1.043e-01 7.745e-02 -1.347 0.1981

SlitOR14:SlitOR25 1.488e-04 6.203e-05 2.399 0.0299 *

SlitOR24:SlitOR25 7.775e-05 5.368e-05 1.449 0.1681

SlitOR7:SlitOR27 -4.209e-03 2.520e-03 -1.670 0.1157

SlitOR14:SlitOR27 -2.373e-03 9.381e-04 -2.529 0.0231 *

SlitOR19:SlitOR27 -1.010e-03 1.052e-03 -0.960 0.3521

SlitOR24:SlitOR27 2.445e-04 8.761e-05 2.791 0.0137 *

SlitOR25:SlitOR27 3.142e-04 2.210e-04 1.422 0.1756

SlitOR7:SlitOR28 -1.291e-02 1.290e-02 -1.001 0.3328

SlitOR24:SlitOR28 6.245e-05 4.259e-05 1.466 0.1632

SlitOR25:SlitOR28 8.198e-06 4.732e-04 0.017 0.9864

SlitOR27:SlitOR28 1.350e-02 8.056e-03 1.675 0.1146

SlitOR7:SlitOR29 -3.082e-03 2.800e-03 -1.101 0.2883

SlitOR24:SlitOR29 9.246e-03 7.674e-03 1.205 0.2469

SlitOR28:SlitOR29 2.728e-04 4.187e-04 0.652 0.5245

SlitOR7:SlitOR14:SlitOR24 8.584e-04 4.268e-04 2.012 0.0626 .

SlitOR7:SlitOR24:SlitOR25 2.994e-04 2.167e-04 1.382 0.1873

SlitOR7:SlitOR25:SlitOR27 7.615e-04 5.636e-04 1.351 0.1966

SlitOR24:SlitOR25:SlitOR27 -6.086e-06 3.133e-06 -1.943 0.0711 .

SlitOR7:SlitOR24:SlitOR28 7.475e-05 7.358e-05 1.016 0.3257

SlitOR24:SlitOR27:SlitOR28 4.452e-04 2.935e-04 1.517 0.1501

SlitOR25:SlitOR27:SlitOR28 -1.539e-04 8.989e-05 -1.712 0.1075

SlitOR24:SlitOR28:SlitOR29 -5.307e-05 3.980e-05 -1.333 0.2023

---

Signif. codes: 0 ‘*’ 0.05 ‘.’ 0.1 ‘ ’ 1

Refined 1:1 Model :

Formula :

PI = SlitOR7 + SlitOR14 + SlitOR24 + SlitOR25 + SlitOR27 + SlitOR7:SlitOR14 + SlitOR7:SlitOR24 + SlitOR14:SlitOR24 + SlitOR7:SlitOR25 + SlitOR14:SlitOR25 + SlitOR24:SlitOR25 + SlitOR7:SlitOR27 + SlitOR24:SlitOR27 + SlitOR25:SlitOR27 + SlitOR7:SlitOR14:SlitOR24 + SlitOR7:SlitOR24:SlitOR25 + SlitOR14:SlitOR24:SlitOR25 + SlitOR7:SlitOR24:SlitOR27 + SlitOR7:SlitOR25:SlitOR27 + SlitOR24:SlitOR25:SlitOR27

Coefficients:

Estimate Std. Error t value Pr(>|t|)

(Intercept) -2.425e-02 2.636e-02 -0.920 0.3648

SlitOR7 1.647e-02 1.947e-02 0.846 0.4041

SlitOR14 -7.345e-03 4.017e-03 -1.828 0.0771 .

SlitOR24 1.560e-03 7.440e-04 2.097 0.0442 *

SlitOR25 3.263e-04 7.166e-04 0.455 0.6520

SlitOR27 7.376e-04 4.033e-04 1.829 0.0771 .

SlitOR7:SlitOR14 -8.105e-03 4.285e-03 -1.892 0.0679 .

SlitOR7:SlitOR24 -7.369e-05 1.545e-04 -0.477 0.6368

SlitOR14:SlitOR24 7.257e-04 4.213e-04 1.722 0.0950 .

SlitOR7:SlitOR25 -1.133e-03 4.989e-04 -2.271 0.0303 *

SlitOR14:SlitOR25 6.087e-05 3.925e-05 1.551 0.1311

SlitOR24:SlitOR25 3.042e-05 1.992e-05 1.527 0.1368

SlitOR7:SlitOR27 -1.250e-04 1.270e-04 -0.984 0.3328

SlitOR24:SlitOR27 2.067e-04 8.154e-05 2.536 0.0165 *

SlitOR25:SlitOR27 3.395e-05 2.818e-05 1.205 0.2374

SlitOR7:SlitOR14:SlitOR24 1.416e-04 7.710e-05 1.837 0.0758 .

SlitOR7:SlitOR24:SlitOR25 4.638e-06 2.064e-06 2.248 0.0318 *

SlitOR14:SlitOR24:SlitOR25 -5.676e-06 3.157e-06 -1.798 0.0819 .

SlitOR7:SlitOR24:SlitOR27 -1.416e-05 7.892e-06 -1.794 0.0825 .

SlitOR7:SlitOR25:SlitOR27 2.071e-05 9.129e-06 2.268 0.0304 *

SlitOR24:SlitOR25:SlitOR27 -2.768e-06 1.123e-06 -2.466 0.0194 *

---

Signif. codes: 0 ‘*’ 0.05 ‘.’ 0.1 ‘ ’ 1

Minimal 1:1 Model :

Formula :

PI = SlitOR7 + SlitOR24 + SlitOR25 + SlitOR27

Coefficients:

Estimate Std. Error t value Pr(>|t|)

(Intercept) -0.0395250 0.0212348 -1.861 0.06896 .

SlitOR7 -0.0050528 0.0023939 -2.111 0.04014 *

SlitOR24 0.0026070 0.0003712 7.024 7.52e-09 ***

SlitOR25 0.0006588 0.0002091 3.151 0.00283 **

SlitOR27 0.0009593 0.0003522 2.724 0.00903 **

---

Signif. codes: 0 ‘***’ 0.001 ‘**’ 0.01 ‘*’ 0.05 ‘.’ 0.1 ‘ ’ 1

1:1/10 Model :

Formula :

PI = SlitOR7 + SlitOR14 + SlitOR24 + SlitOR25 + SlitOR27 + SlitOR28 + SlitOR29 + SlitOR24:SlitOR25 + SlitOR24:SlitOR28 + SlitOR24:SlitOR29 + SlitOR28:SlitOR29 + SlitOR24:SlitOR28:SlitOR29

Coefficients:

Estimate Std. Error t value Pr(>|t|)

(Intercept) -1.987e-02 2.405e-02 -0.826 0.41362

SlitOR7 -5.410e-02 3.149e-02 -1.718 0.09347 .

SlitOR14 -5.363e-04 3.574e-04 -1.501 0.14128

SlitOR24 4.648e-03 7.784e-04 5.971 5.20e-07 ***

SlitOR25 1.706e-03 3.607e-04 4.729 2.79e-05 ***

SlitOR27 6.204e-04 3.911e-04 1.587 0.12049

SlitOR28 -3.667e-04 3.246e-03 -0.113 0.91062

SlitOR29 6.906e-04 5.161e-04 1.338 0.18843

SlitOR24:SlitOR25 -2.009e-05 6.588e-06 -3.049 0.00406 **

SlitOR24:SlitOR28 -1.490e-05 3.472e-05 -0.429 0.67009

SlitOR24:SlitOR29 -2.714e-02 9.109e-03 -2.980 0.00489 **

SlitOR28:SlitOR29 9.573e-05 3.299e-04 0.290 0.77317

SlitOR24:SlitOR28:SlitOR29 1.408e-04 4.649e-05 3.028 0.00429 **

---

Signif. codes: 0 ‘***’ 0.001 ‘**’ 0.01 ‘.’ 0.1 ‘ ’ 1

1:1/100 Model :

Formula :

PI = SlitOR7 + SlitOR14 + SlitOR19 + SlitOR24 + SlitOR25 + SlitOR27 + SlitOR28 + SlitOR29 + SlitOR31 + SlitOR14:SlitOR25 + SlitOR19:SlitOR25 + SlitOR24:SlitOR25 + SlitOR24:SlitOR27 + SlitOR25:SlitOR27 + SlitOR24:SlitOR28 + SlitOR25:SlitOR28 + SlitOR27:SlitOR28 + SlitOR24:SlitOR29 + SlitOR28:SlitOR29 + SlitOR24:SlitOR25:SlitOR28 + SlitOR24:SlitOR27:SlitOR28 + SlitOR24:SlitOR28:SlitOR29

Coefficients:

Estimate Std. Error t value Pr(>|t|)

(Intercept) -3.413e-02 2.675e-02 -1.276 0.211815

SlitOR7 9.592e-02 3.975e-02 2.413 0.022145 *

SlitOR14 3.027e-04 8.538e-04 0.355 0.725416

SlitOR19 -1.742e-02 2.578e-02 -0.676 0.504418

SlitOR24 1.092e-02 2.013e-03 5.426 7e-06 ***

SlitOR25 2.245e-03 3.517e-03 0.638 0.528082

SlitOR27 8.294e-04 5.243e-04 1.582 0.124152

SlitOR28 3.962e-03 4.949e-03 0.800 0.429727

SlitOR29 1.066e-03 8.396e-04 1.270 0.213924

SlitOR31 3.268e-03 3.029e-03 1.079 0.289350

SlitOR14:SlitOR25 -6.291e-05 3.742e-05 -1.681 0.103115

SlitOR19:SlitOR25 9.179e-04 6.285e-04 1.460 0.154548

SlitOR24:SlitOR25 -7.381e-05 5.753e-05 -1.283 0.209267

SlitOR24:SlitOR27 -5.786e-03 2.384e-03 -2.427 0.021423 *

SlitOR25:SlitOR27 4.532e-03 2.379e-03 1.905 0.066375 .

SlitOR24:SlitOR28 -1.794e-04 4.645e-05 -3.863 0.000555 ***

SlitOR25:SlitOR28 1.244e-02 7.113e-03 1.748 0.090629 .

SlitOR27:SlitOR28 -5.837e-01 3.037e-01 -1.922 0.064150 .

SlitOR24:SlitOR29 1.270e-01 4.839e-02 2.625 0.013510 *

SlitOR28:SlitOR29 -2.691e-03 1.214e-03 -2.218 0.034308 *

SlitOR24:SlitOR25:SlitOR28 -4.379e-03 2.419e-03 -1.810 0.080265 .

SlitOR24:SlitOR27:SlitOR28 1.287e-01 6.844e-02 1.881 0.069721 .

SlitOR24:SlitOR28:SlitOR29 -3.601e-04 1.427e-04 -2.524 0.017119 *

---

Signif. codes: 0 ‘***’ 0.001 ‘*’ 0.05 ‘.’ 0.1 ‘ ’ 1

1:1/1000 Model :

Formula :

PI = SlitOR24 + SlitOR25 + SlitOR27 + SlitOR28 + SlitOR29 + SlitOR24:SlitOR27 + SlitOR25:SlitOR27 + SlitOR27:SlitOR28 + SlitOR24:SlitOR29 + SlitOR28:SlitOR29

Coefficients:

Estimate Std. Error t value Pr(>|t|)

(Intercept) 0.0375190 0.0246582 1.522 0.13561

SlitOR24 0.0113425 0.0032187 3.524 0.00104 **

SlitOR25 -0.0011517 0.0035231 -0.327 0.74536

SlitOR27 0.0003768 0.0009738 0.387 0.70079

SlitOR28 -0.0149559 0.0043652 -3.426 0.00138 **

SlitOR29 -0.0001596 0.0020459 -0.078 0.93818

SlitOR24:SlitOR27 -0.0062024 0.0023431 -2.647 0.01138 *

SlitOR25:SlitOR27 0.0055193 0.0022047 2.503 0.01628 *

SlitOR27:SlitOR28 -0.0359399 0.0154458 -2.327 0.02487 *

SlitOR24:SlitOR29 -0.0519243 0.0358747 -1.447 0.15521

SlitOR28:SlitOR29 0.0030420 0.0013116 2.319 0.02532 *

---

Signif. codes: 0 ‘**’ 0.01 ‘*’ 0.05 ‘.’ 0.1 ‘ ’ 1
